# Dramatically elevated plasma vascular endothelial growth factor levels from influenza A infection in polyneuropathy, organomegaly, endocrinopathy, monoclonal gammopathy, and skin changes syndrome: A case report

**DOI:** 10.1002/jha2.965

**Published:** 2024-06-12

**Authors:** Ashwin Kannan, Ying Zhuo, Rahul Banerjee

**Affiliations:** ^1^ Clinical Research Division Fred Hutchinson Cancer Center Seattle Washington USA; ^2^ Hematology/Oncology Kadlec Clinic Kennewick Washington USA

**Keywords:** influenza, multiple myeloma, POEMS, VEGF

## Abstract

We present a patient with polyneuropathy, organomegaly, endocrinopathy, monoclonal gammopathy, and skin changes (POEMS) syndrome who had a dramatic and sustained elevation in plasma vascular endothelial growth factor (VEGF) levels from 182 to 740 pg/mL while on lenalidomide‐dexamethasone therapy. Given his biochemical evidence of progression, second‐line daratumumab was added. In hindsight, a concurrent influenza A infection was the likely driver of his VEGF elevation rather than his underlying POEMS syndrome. Given the importance of longitudinal VEGF monitoring and the infectious risks of plasma cell therapies, our case highlights the need for caution with POEMS response assessments in the setting of a respiratory viral infection.

## 1 INTRODUCTION

POEMS syndrome (polyneuropathy, organomegaly, endocrinopathy, monoclonal gammopathy, and skin changes) is a rare paraneoplastic condition associated with plasma cell disorders. The diagnosis of POEMS syndrome requires at least three major criteria, one of which can be the elevation of vascular endothelial growth factor (VEGF) levels [1]. A plasma VEGF level of 200 pg/mL is very sensitive for POEMS syndrome and is unlikely to be seen in other plasma cell disorders [2]. For patients with POEMS syndrome, a plasma VEGF elevation exceeding 50% of its lowest value represents progressive disease [1].

We present the case of a 56‐year‐old male with POEMS syndrome whose case is unique in two respects. Firstly, his neuropathy onset was more rapid than usual due to concomitant vitamin B12 deficiency. Secondly and more importantly, several months after initiating treatment with lenalidomide and dexamethasone (Rd), he experienced a marked elevation in his plasma VEGF level from a nadir of 182–740 pg/mL over 2 months without any worsening of his neuropathy. Second‐line daratumumab was immediately added as a precaution; however, in hindsight, a concurrent influenza A diagnosis may have been the true driver of his VEGF elevation. Given the importance of longitudinal VEGF monitoring to POEMS syndrome coupled with the risk of infections inherent to plasma cell‐directed therapies, we hope this case will raise awareness of this potential association for clinicians who care for these patients.

## 2 CASE REPORT

A 56‐year‐old male presented with a 2‐month history of worsening symmetric leg numbness and multiple falls. Medical history included coronary artery disease; medications and social history were non‐contributory. Physical exam showed decreased ankle jerk reflexes and foot drop bilaterally. Vitamin B12 level was 166 pg/mL (lower limit of normal 232 pg/mL), serum protein electrophoresis showed a biclonal immunoglobulin A (IgA) M‐spike of 1.1 and 0.3, serum kappa free light chain (FLC) was 37.3 milligrams per liter (mg/L) with upper limit of normal (ULN) 19.4 mg/L, lambda FLC 188.0 mg/L (ULN 26.3 mg/L), FLC ratio 0.20, and plasma VEGF 356 pg/mL. He had no myeloma‐defining events. Bone marrow biopsy showed 25%–30% plasma cells, while cross‐sectional imaging revealed only an osteosclerotic 9th rib lesion.

**FIGURE 1:**
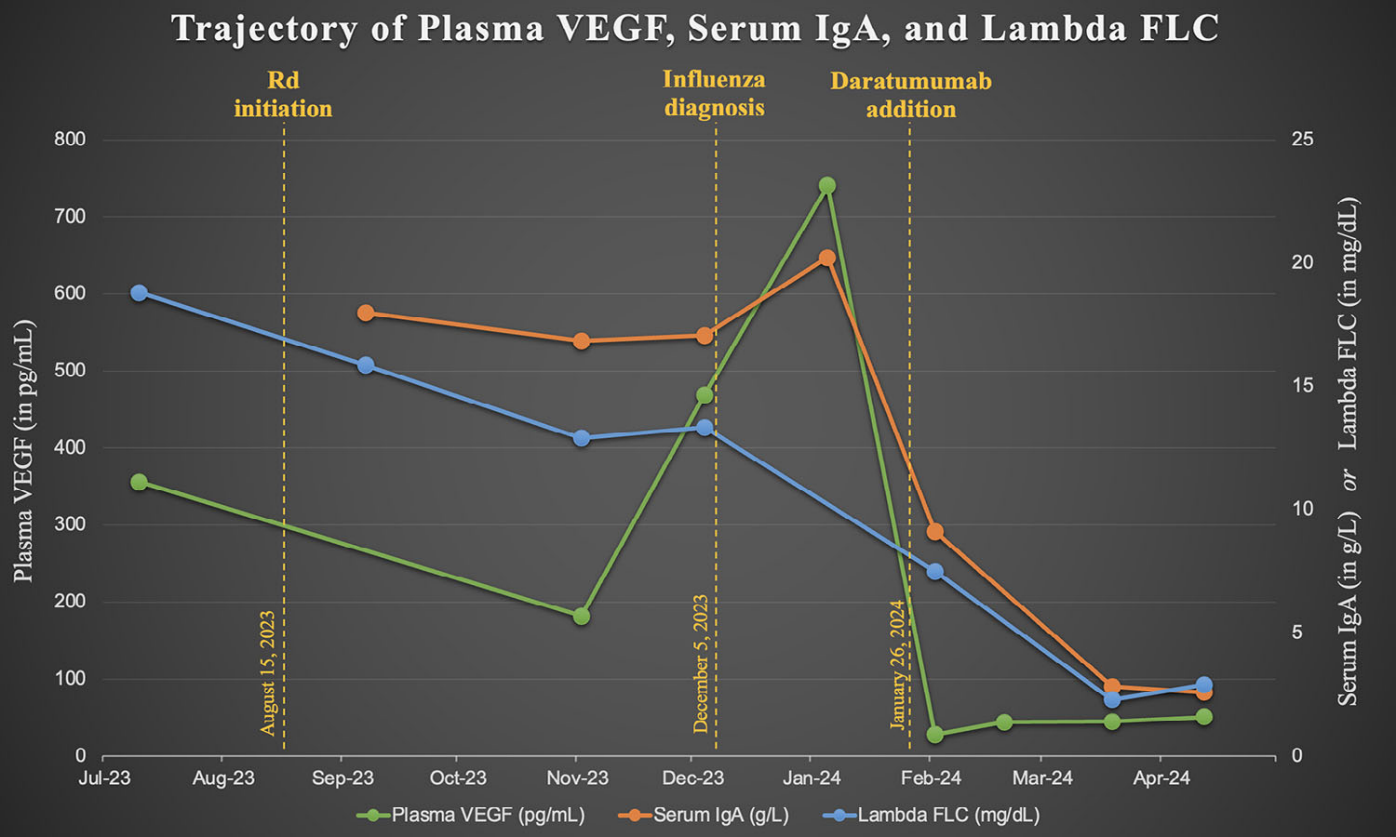
Trajectory of plasma VEGF, serum immunoglobulin A (IgA), and lambda FLC. *[uploaded separately]*. Units for serum IgA and lambda FLC are converted from their description in the text for ease of viewing values on the same axis. FLC, free light chain (serum); g/L, grams per liter; mg/dL, milligrams per deciliter; pg/mL, picograms per milliliter; Rd, lenalidomide dexamethasone; VEGF, vascular endothelial growth factor.

He was started on Rd doublet therapy (Figure 1) with a 49% reduction in his plasma VEGF from 356 to 182 pg/mL after four 28‐day cycles. With concurrent cyanocobalamin repletion, his vitamin B12 level rose from 166 to 1389 pg/mL. The patient reported dramatic improvement in his neuropathy with these interventions. Before his fifth Rd cycle, the patient presented for routine pre‐cycle bloodwork and endorsed mild new‐onset respiratory symptoms. Lambda FLC was 133.3 mg/L and serum IgA 1706 milligrams per deciliter (mg/dL), both of which were roughly stable; however, his plasma VEGF had more than doubled to 469 pg/mL.

The following day, the patient's symptoms worsened and he was diagnosed with influenza A at an urgent care clinic. He was started on oseltamivir and his POEMS therapy was delayed by 1 month, at which point plasma VEGF was 740 pg/mL (a fourfold increase over its value 2 months earlier). Lambda FLC was not checked due to a laboratory error, but serum IgA had increased only mildly to 2022 mg/dL at this time (Figure 1 ). The patient reported the resolution of his influenza symptoms by this point and denied any interval worsening of his neuropathy. Given his rapidly rising VEGF level and his previously severe neurological symptoms, however, subcutaneous daratumumab with hyaluronidase‐fihj was urgently added after shared decision‐making. After one cycle of daratumumab plus Rd, his plasma VEGF normalized to 28 pg/mL before stabilizing in the 40–60 pg/mL range. His IgA and lambda FLC levels remain normal, and he continues to feel well.

## 3 DISCUSSION

In brief, we report a patient with POEMS syndrome whose VEGF level rose from 182 to 469 pg/mL one day before his clinical diagnosis with influenza A. His VEGF subsequently rose as high as 740 pg/mL, constituting a 4‐fold elevation within 2 months. Importantly, the patient's neuropathy had previously improved with the initiation of Rd therapy and did not worsen at all during this period. This suggests that influenza, not POEMS progression, was the driver of his marked VEGF elevation. Other examples of changes in VEGF levels irrespective of POEMS activity, for example, in the setting of intravitreal anti‐VEGF injections, have recently been reported as well [3].

Strengths of the putative association between influenza A and elevated VEGF levels include previous studies demonstrating that elevated VEGF levels during influenza infections are associated with worsened morbidity [4, 5]. Interestingly, our patient only had moderate influenza symptoms managed with oseltamivir in the outpatient setting. Our patient's VEGF level was elevated immediately before his influenza diagnosis, likely during the viral prodrome period. Arguing against a POEMS‐related explanation is his relatively stable serum IgA level and his lack of worsening neurological symptoms. The fact that his VEGF levels peaked 1 month after influenza diagnosis may reflect exaggerated VEGF production in response to inflammation in patients with POEMS syndrome; additionally, peak serum VEGF elevations have been shown to lag behind influenza A virus infection in animal models [6].

One unanswered question is whether, if we had taken no action beyond oseltamivir and resuming Rd, his VEGF level would have fallen with time. Given the severity of his initial neurologic presentation and his clear evidence of progression by POEMS criteria [1], we opted to add second‐line daratumumab with rapid VEGF and light chain normalization thereafter. Daratumumab is an effective treatment modality for POEMS syndrome but, even more so than lenalidomide, does carry an increased risk of viral infections [7, 8]. Other viral infections such as the severe acute respiratory syndrome coronavirus 2 can increase VEGF levels as well [9, 10], although we are not aware of any case reports of this phenomenon with coronavirus disease 2019. Nevertheless, as daratumumab and other novel therapies gain adoption in the management of POEMS syndrome, we advise caution with POEMS response assessments in the setting of a respiratory viral infection.

## AUTHOR CONTRIBUTIONS

Ashwin Kannan and Rahul Banerjee wrote the first draft. All authors provided critical feedback and approved the final manuscript.

## CONFLICT OF INTEREST STATEMENT

Rahul Banerjee reports consulting: Adaptive, BMS, Caribou Biosciences, Genentech, Janssen, Karyopharm, Legend Biotech, Pfizer, Sanofi, SparkCures; Research: Novartis, and Pack Health. The remaining authors declare no conflict of interest.

## FUNDING INFORMATION

The authors received no specific funding for this work.

## ETHICS STATEMENT

The authors have confirmed ethical approval statement is not needed for this submission.

## PATIENT CONSENT STATEMENT

The patient provided his consent for this case report's publication.

## CLINICAL TRIAL REGISTRATION

The authors have confirmed clinical trial registration is not needed for this submission.

## Data Availability

Not available.
